# Supplementary material for: A dispensable paralog of succinate dehydrogenase subunit C mediates standing resistance towards a subclass of SDHI fungicides in Zymoseptoria tritici
Source: PLoS Pathog. 2019 Dec 20;15(12):e1007780. doi: 10.1371/journal.ppat.1007780 (PMC6941823; doi:10.1371/journal.ppat.1007780)

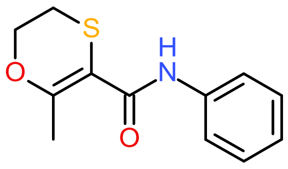

Carboxin

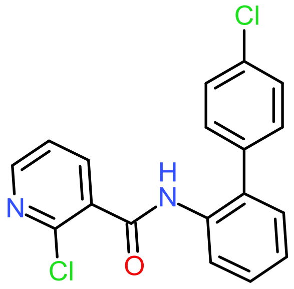

Boscalid

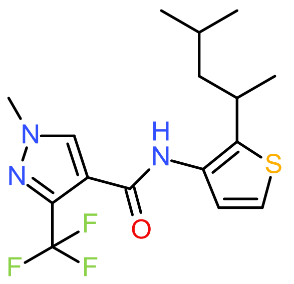

Penthiopyrad

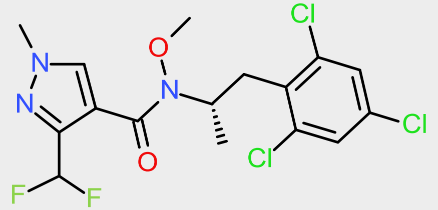

Pydiflumetofen

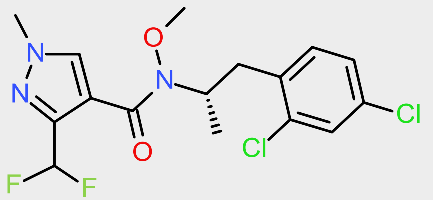

Compound 3

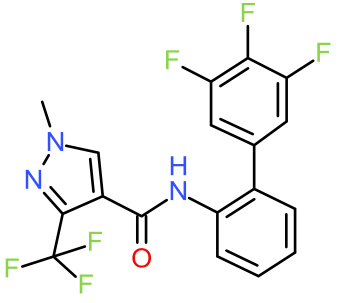

Fluxapyroxad

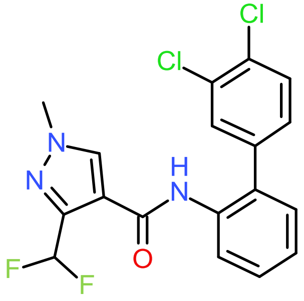

Bixafen

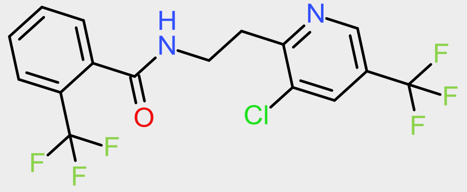

Fluopyram

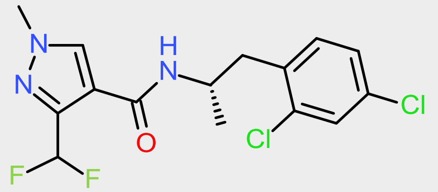

Compound 2

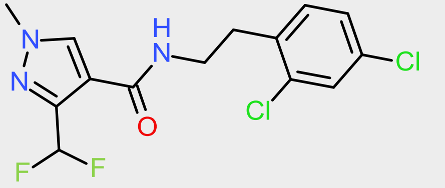

Compound 1

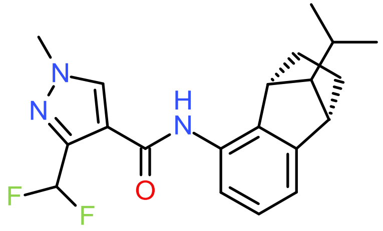

Isopyrazam

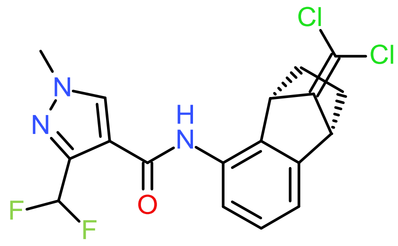

Benzovindiflupyr

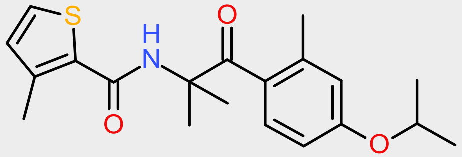

Isofetamid

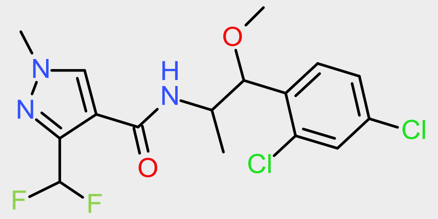

Compound 4

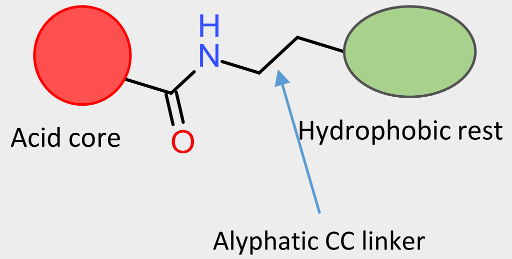

Supplement: S1 Fig — Shaded grey area represents the SHA cross-resistance group. Schematic view of a typical SHA compound is shown in the bottom right corner. (PDF) [file ppat.1007780.s001.pdf]
