# Supplementary figures and images for: A dispensable paralog of succinate dehydrogenase subunit C mediates standing resistance towards a subclass of SDHI fungicides in Zymoseptoria tritici
Source: PLoS Pathog. 2019 Dec 20;15(12):e1007780. doi: 10.1371/journal.ppat.1007780 (PMC6941823; doi:10.1371/journal.ppat.1007780)

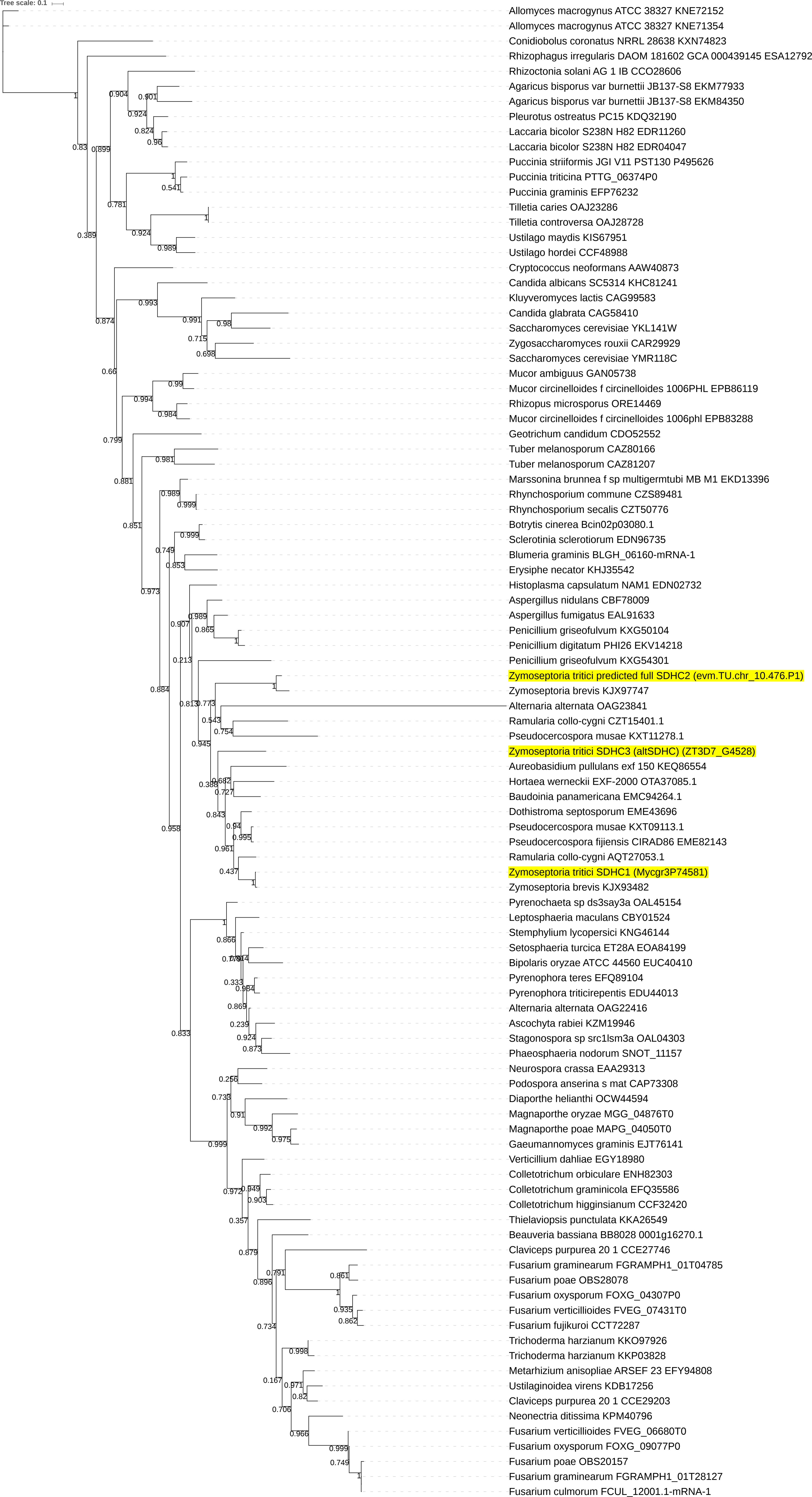

Supplement: S2 Fig — Tree generated using PhyML and visualized using iTOL (see material and methods). ZtSDHC1-3 paralogs are highlighted in yellow. (PDF) [file ppat.1007780.s002.pdf]

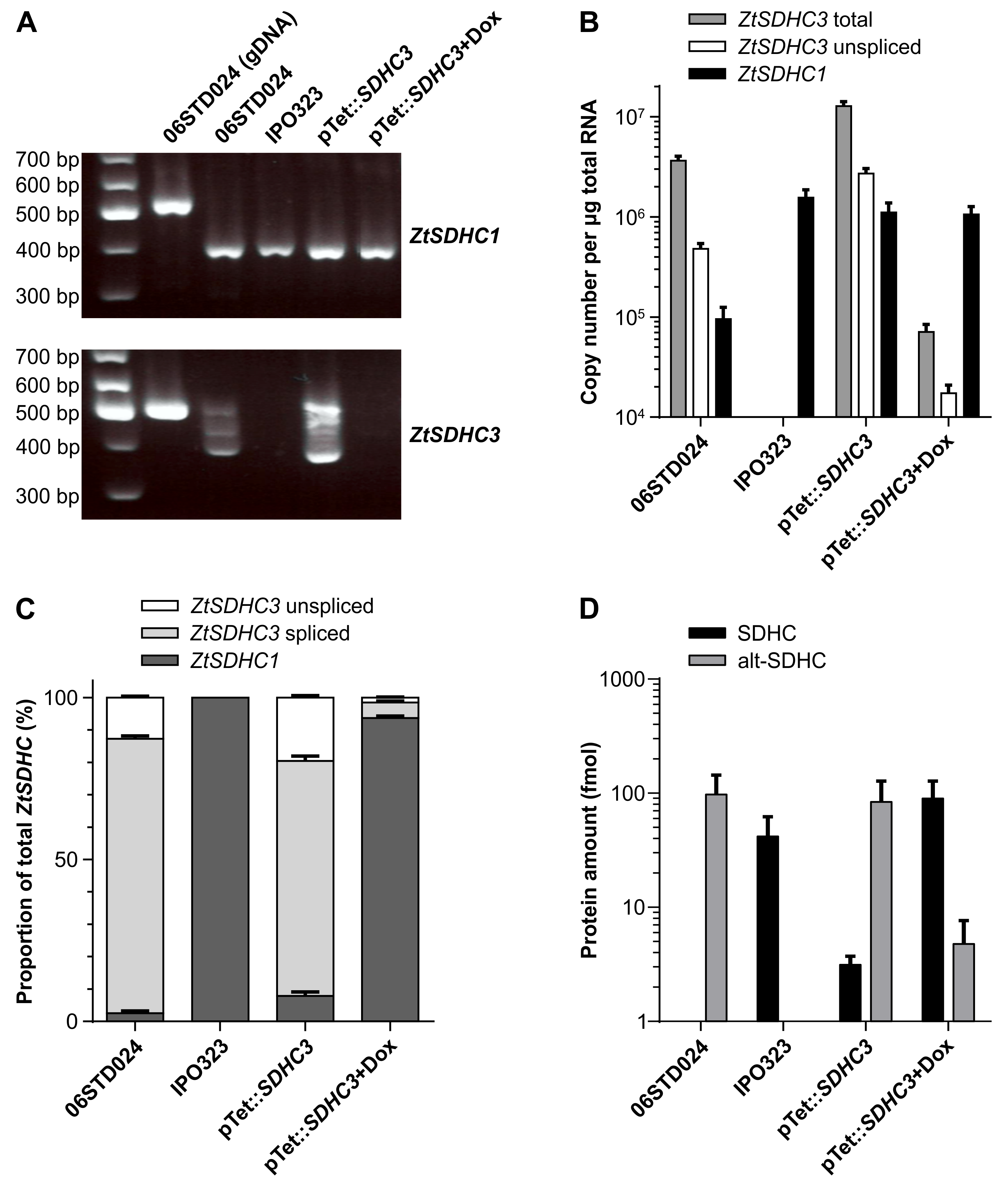

Supplement: S5 Fig — (A) RT-PCR analysis of SDHC1 and ZtSDHC3 in 06STD024 and IPO323 pTet:SDHC3 transformant. The expected PCR products corresponding to fully spliced mRNAs were 389 and 384 bp for ZtSDHC1 and ZtSDHC3 respectively. (B) Absolute quantification by RT-qPCR of the three SDHC mRNA species in the 06STD024 strain and IPO323 pTet::SDHC3 transformant. (C) Normalized proportion of the three mRNA species (as deducted from panel B). (D) LC-MS/MS quantification of the SDHC and alt-SDHC proteins in mitochondrial extracts from 06STD024 and IPO323 pTet::SDHC3 transformant. Values presented are the mean of 6 individual experiments ± SD. (TIF) [file ppat.1007780.s005.tif]

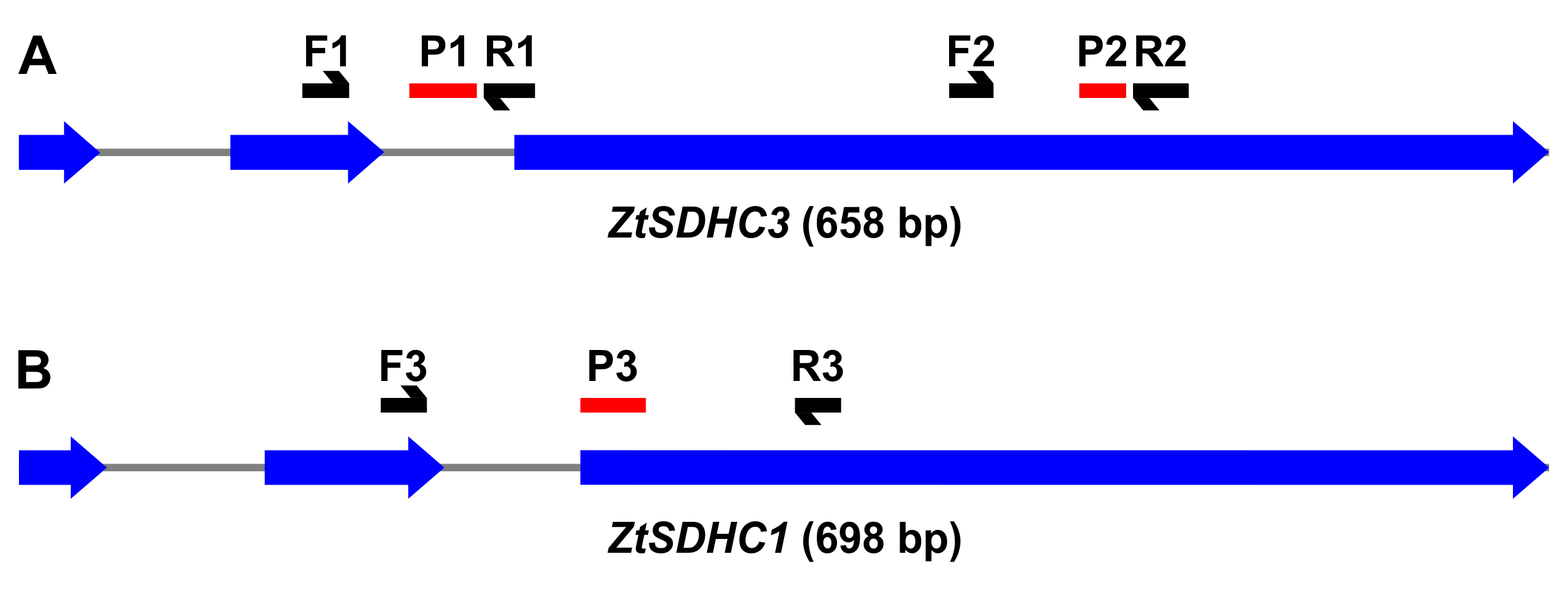

Supplement: S6 Fig — Exons are shown as blue arrows and introns as grey bars, labelled hydrolysis probes are shown in red, forward and reverse PCR oligos are shown as black arrows. Oligonucleotides sequences and probe details are shown in S4 Dataset. (TIF) [file ppat.1007780.s006.tif]
