# Supplementary material for: A dispensable paralog of succinate dehydrogenase subunit C mediates standing resistance towards a subclass of SDHI fungicides in Zymoseptoria tritici
Source: PLoS Pathog. 2019 Dec 20;15(12):e1007780. doi: 10.1371/journal.ppat.1007780 (PMC6941823; doi:10.1371/journal.ppat.1007780)
Supplement: S4 Table — (DOCX) [file ppat.1007780.s010.docx]

| **Species** | **Predicted SDHC paralogs** |
| --- | --- |
| **Agaricus bisporus var burnettii JB137-S8** | **2** |
| **Allomyces macrogynus ATCC 38327** | **2** |
| **Alternaria alternata** | **2** |
| Ascochyta rabiei | 1 |
| Aspergillus fumigatus | 1 |
| Aspergillus nidulans | 1 |
| Aureobasidium pullulans exf 150 | 1 |
| Baudoinia panamericana | 1 |
| Beauveria bassiana BB8028 | 1 |
| Bipolaris oryzae ATCC 44560 | 1 |
| Blumeria graminis | 1 |
| Botrytis cinerea | 1 |
| Candida albicans SC5314 | 1 |
| Candida glabrata | 1 |
| **Claviceps purpurea 20 1** | **2** |
| Colletotrichum graminicola | 1 |
| Colletotrichum higginsianum | 1 |
| Colletotrichum orbiculare | 1 |
| Conidiobolus coronatus NRRL 28638 | 1 |
| Cryptococcus neoformans | 1 |
| Diaporthe helianthi | 1 |
| Dothistroma septosporum | 1 |
| Erysiphe necator | 1 |
| Fusarium culmorum | 1 |
| Fusarium fujikuroi | 1 |
| **Fusarium graminearum** | **2** |
| **Fusarium oxysporum** | **2** |
| **Fusarium poae** | **2** |
| **Fusarium verticillioides** | **2** |
| Gaeumannomyces graminis | 1 |
| Geotrichum candidum | 1 |
| Histoplasma capsulatum | 1 |
| Hortaea werneckii EXF-2000 | 1 |
| Kluyveromyces lactis | 1 |
| **Laccaria bicolor S238N H82** | **2** |
| Leptosphaeria maculans | 1 |
| Magnaporthe oryzae | 1 |
| Magnaporthe poae | 1 |
| Marssonina brunnea f sp multigermtubi MB M1 | 1 |
| Metarhizium anisopliae ARSEF 23 | 1 |
| Mucor ambiguus | 1 |
| **Mucor circinelloides f circinelloides 1006phl** | **2** |
| Neonectria ditissima | 1 |
| Neurospora crassa | 1 |
| Penicillium digitatum PHI26 | 1 |
| **Penicillium griseofulvum** | **2** |
| Phaeosphaeria nodorum | 1 |
| Pleurotus ostreatus PC15 | 1 |
| Podospora anserina s mat | 1 |
| Pseudocercospora fijiensis CIRAD86 | 1 |
| **Pseudocercospora musae** | **2** |
| Puccinia graminis | 1 |
| Puccinia striiformis JGI V11 PST130 | 1 |
| Puccinia triticina | 1 |
| Pyrenochaeta sp ds3say3a | 1 |
| Pyrenophora teres | 1 |
| Pyrenophora triticirepentis | 1 |
| **Ramularia collo-cygni** | **2** |
| Rhizoctonia solani AG 1 IB | 1 |
| Rhizophagus irregularis DAOM 181602 GCA 000439145 | 1 |
| Rhizopus microsporus | 1 |
| Rhynchosporium commune | 1 |
| Rhynchosporium secalis | 1 |
| **Saccharomyces cerevisiae** | **2** |
| Sclerotinia sclerotiorum | 1 |
| Setosphaeria turcica ET28A | 1 |
| Stagonospora sp src1lsm3a | 1 |
| Stemphylium lycopersici | 1 |
| Thielaviopsis punctulata | 1 |
| Tilletia caries | 1 |
| Tilletia controversa | 1 |
| **Trichoderma harzianum** | **2** |
| **Tuber melanosporum** | **2** |
| Ustilaginoidea virens | 1 |
| Ustilago hordei | 1 |
| Ustilago maydis | 1 |
| Verticillium dahliae | 1 |
| Zygosaccharomyces rouxii | 1 |
| **Zymoseptoria brevis** | **2** |
| **Zymoseptoria tritici** | **3** |
