# Supplementary material for: A dispensable paralog of succinate dehydrogenase subunit C mediates standing resistance towards a subclass of SDHI fungicides in Zymoseptoria tritici
Source: PLoS Pathog. 2019 Dec 20;15(12):e1007780. doi: 10.1371/journal.ppat.1007780 (PMC6941823; doi:10.1371/journal.ppat.1007780)
Supplement: S3 Fig — Expression data inferred from RNAseq (Rudd et al., 2015). (PDF) [file ppat.1007780.s003.pdf]

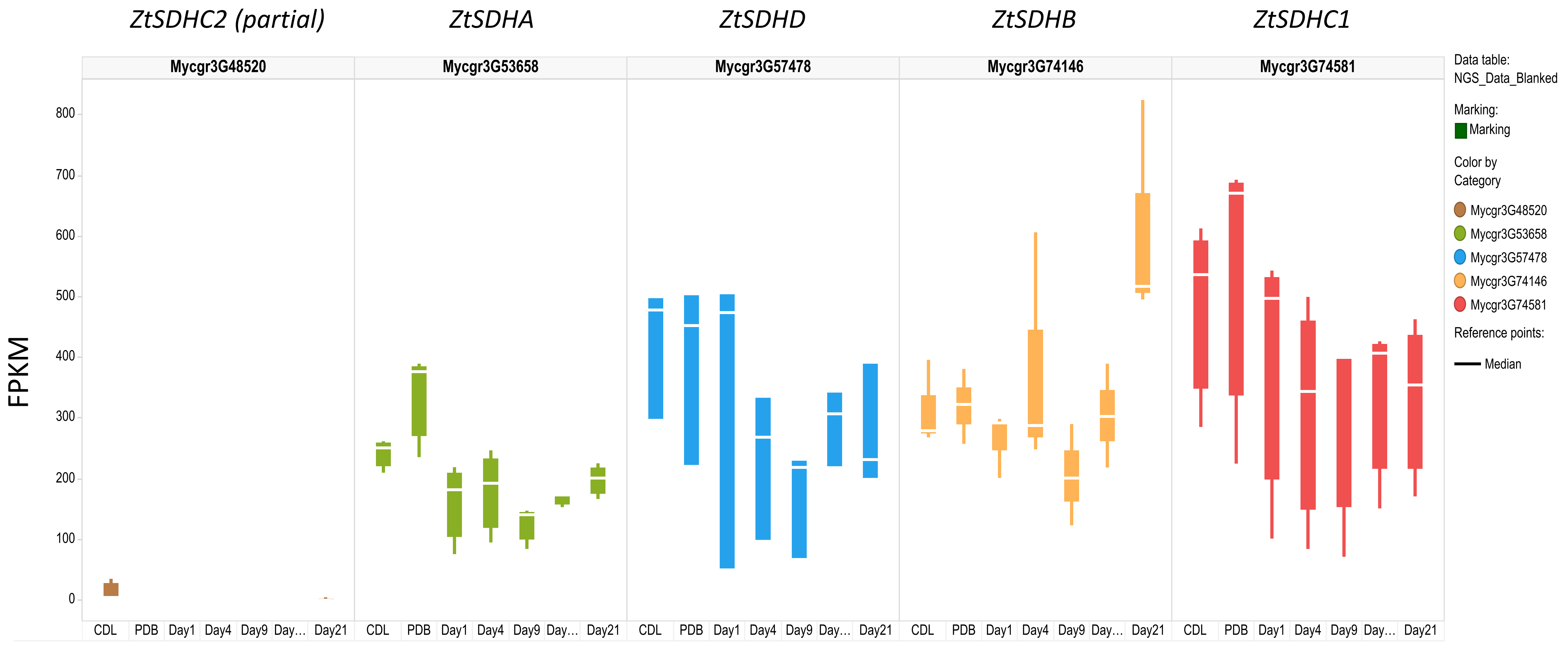
ZtSDHC2 (partial)
ZtSDHA
ZtSDHD
ZtSDHB
ZtSDHC1
Mycgr3G48520
Mycgr3G53658
Mycgr3G57478
Mycgr3G74146
Mycgr3G74581
FPKM
0
100
200
300
400
500
600
700
800
CDL
PDB
Day1
Day4
Day9
Day…
Day21
Data table:
NGS_Data_Blanked
Marking:
Marking
Color by
Category
Mycgr3G48520
Mycgr3G53658
Mycgr3G57478
Mycgr3G74146
Mycgr3G74581
Reference points:
Median
